# Multifunctional Effects of Peach Palm (*Bactris gasipaes*) Heart Powder on the Printability, Structural Properties, Probiotic Viability, and Gastrointestinal Digestion of Soy Protein-Based 3D-Printed Foods

**DOI:** 10.3390/foods15183336

**Published:** 2026-09-20

**Authors:** Passakorn Kingwascharapong, Jaksuma Pongsetkul, Supatra Karnjanapratum, Pittaya Chaikham, Saroat Rawdkuen, Sani Jirasatid, Samart Sai-Ut

**Affiliations:** 1 Department of Fishery Products, Faculty of Fisheries, Kasetsart University, Bangkok 10900, Thailand; passakorn.ki@ku.th; 2 School of Animal Technology and Innovation, Institute of Agricultural Technology, Suranaree University of Technology, Nakhon Ratchasima 30000, Thailand; jaksuma@sut.ac.th; 3 Faculty of Agro-Industry, Chiang Mai University, Chiang Mai 50200, Thailand; supatra.ka@cmu.ac.th; 4 Division of Food Science and Technology Management, Faculty of Science and Technology, Phranakhon Si Ayutthaya Rajabhat University, Phranakhon Si Ayutthaya 13000, Thailand; pittaya.chaikham@gmail.com; 5 Food Science and Technology Program, School of Agro-Industry, Mae Fah Luang University, Chiang Rai 57100, Thailand; saroat@mfu.ac.th; 6 Department of Food Science, Faculty of Science, Burapha University, Chonburi 20131, Thailand; sani@go.buu.ac.th

**Keywords:** 3D printing, peach palm heart, structural properties, digestibility, bioink

## Abstract

Peach palm (*Bactris gasipaes*) heart is a sustainable, fiber- and polysaccharide-rich ingredient with potential for plant-based food structuring. This study investigated peach palm heart powder (PPHP) as a functional co-ingredient in soy protein isolate (SPI)-based inks for extrusion-based 3D food printing, focusing on rheology, printability, microstructure, molecular interactions, texture, and *in vitro* digestibility. Increasing PPHP concentration enhanced apparent and complex viscosity, storage modulus (*G*′), deformation resistance, and structural recovery while maintaining strong shear-thinning behavior, improving printing performance; PPHP8 showed the lowest dimensional deviation (0.25%) and highest shape fidelity (100.25%). PPHP was progressively incorporated into the SPI matrix, forming a denser protein–polysaccharide network primarily through non-covalent interactions, as indicated by microstructural and SDS–PAGE analyses. PPHP reduced hardness, gumminess, chewiness, and resilience but maintained springiness, enabling texture modulation, and FTIR/PCA revealed a shift from α-helical toward β-sheet-rich protein conformations, consistent with enhanced hydrogen bonding. Protein hydrolysis during simulated digestion and *B. longum* subsp. *longum* viability were not significantly compromised by PPHP incorporation. These findings demonstrate that PPHP can be strategically incorporated to tailor the rheological and textural properties of SPI-based inks for 3D printing without compromising nutritional functionality, supporting its potential as a sustainable structuring ingredient for next-generation plant-based printed foods.

## 1. Introduction

Peach palm (*Bactris gasipaes*) is an emerging economic crop in Thailand, valued for its adaptability to tropical climates, high productivity, and potential in value-added food applications [1,2]. Although traditionally cultivated for its edible fruit and palm heart, nearly every part of the plant can be utilized, supporting sustainable production with minimal waste [3]. The fruit is particularly rich in carbohydrates, dietary fiber, carotenoids, vitamins, and minerals, while peach palm flour contains abundant non-starch polysaccharides with high water-binding and thickening capacities. In addition, peach palm heart and its by-products are rich in structural polysaccharides, primarily cellulose and hemicelluloses (e.g.xylans). These by-products contain approximately 34% cellulose and 21% hemicellulose (dry weight), making them a promising source of functional dietary fiber for food applications [1]. Collectively, these compositional and functional characteristics highlight the potential of peach palm heart-derived fiber-rich ingredients as novel food ingredients; however, their application in advanced processing technologies such as three-dimensional (3D) food printing remains largely unexplored.

Extrusion-based 3D food printing enables precise, layer-by-layer deposition of edible materials to fabricate customized structures for personalized nutrition, elderly-friendly foods, and plant-based products [4,5]. Its success, however, depends critically on the rheological behavior of the food ink: the material must exhibit sufficient shear-thinning behavior to allow smooth extrusion while recovering its structure sufficiently rapidly to preserve shape fidelity after deposition. Developing food inks with this balance between extrudability and post-deposition structural stability remains a central challenge in the field.

Soy protein isolate (SPI) is widely used in 3D-printed foods owing to its high protein content, balanced amino acid profile, and gel-forming ability [6,7], but SPI alone often lacks the mechanical strength and structural stability needed for accurate printing. Hydrocolloids such as xanthan gum, carrageenan, alginate, pectin, cellulose derivatives, and starches are commonly incorporated to improve viscosity and gel strength through hydrogen bonding, electrostatic interactions, and physical entanglement with protein molecules [8,9]. Several studies have demonstrated the beneficial effects of polysaccharides on the printability of SPI-based food inks. Wang et al. [10] reported that incorporating 1.5% carrageenan and 1.5% sodium alginate significantly improved the hardness, adhesiveness, apparent viscosity, and storage modulus (*G*′) of the paste, thereby enhancing the printability and quality of 3D-printed products. Similarly, Liu et al. [11] demonstrated that Tremella polysaccharides and psyllium husk powder improved the printability and swallowing properties of SPI gels for dysphagia-friendly foods. Likewise, Xu et al. [12] found that *Naematelia aurantialba* polysaccharides dose-dependently enhanced the rheological properties, gel strength, and 3D printability of SPI gels.

Peach palm heart powder (PPHP), owing to its dietary fiber and polysaccharide components and associated water-binding and thickening properties, may represent a promising and sustainable functional co-ingredient for SPI-based food inks while simultaneously adding value to an underutilized crop. Beyond their technological role, dietary fibers and polysaccharide components within PPHP may interact with proteins and thereby modify gel network architecture, water distribution, and mechanical properties, with downstream effects on enzyme accessibility and protein hydrolysis kinetics during gastrointestinal digestion. Previous work by Cantu-Jungles et al. [13] demonstrated that pectins extracted and purified from peach palm fruit were readily fermented by the human gut microbiota and produced significantly less gas than fructooligosaccharides, suggesting potential functionality as a dietary fiber. However, this evidence was obtained using an isolated peach palm pectin fraction rather than whole PPHP. Therefore, these findings provide a rationale for investigating the potential functionality of peach palm-derived fiber in a food matrix, but they cannot be directly extrapolated to PPHP or interpreted as evidence of a prebiotic effect of the whole powder. Whether PPHP can support the growth of beneficial intestinal bacteria therefore requires direct evaluation using the whole ingredient. In addition, the incorporation of PPHP into protein-based food matrices may alter the structural organization of the gel and consequently influence protein accessibility to digestive enzymes. However, such structure–digestibility relationships remain poorly understood for PPHP-containing, 3D-printed protein matrices.

This study therefore investigated the effects of PPHP incorporation on the rheological behavior, printability, structural formation, and simulated gastrointestinal digestion of SPI-based 3D-printed foods. Rheological measurements, printing performance, textural analysis, and microstructural characterization were used to evaluate the effects of PPHP incorporation on the physicochemical properties of the printed matrix, while in vitro gastrointestinal digestion was used to assess its effects on protein hydrolysis. In addition, the viability of *Bifidobacterium longum* subsp. *longum* was evaluated to provide preliminary evidence regarding the potential functionality of PPHP-containing formulations for beneficial bacterial growth. The findings aim to clarify the technological and functional effects of incorporating whole PPHP into SPI-based 3D-printed foods and to assess its potential as a sustainable, fiber-rich food ingredient.

## 2. Materials and Methods

### 2.1. Chemicals

PPHP was purchased from Tevada Global Co., Ltd. (Chiang Mai, Thailand). The chemical composition of PPHP was determined prior to its use in the formulation and consisted of 8.47 ± 0.07% moisture, 6.03 ± 0.04% ash, 5.30 ± 0.08% protein, 4.65 ± 0.15% fat, and 18.34 ± 0.14% dietary fiber. The carbohydrate content was 75.27%, calculated by difference. Soybean protein isolate (SPI, ≥90% protein, dry basis) and sodium alginate were purchased from Chanjao Longevity Co., Ltd. (Bangkok, Thailand). 2,4,6-Trinitrobenzenesulfonic acid solution (TNBS) was purchased from Sigma-Aldrich Chemical (St. Louis, MO, USA). Sodium phosphate dibasic heptahydrate (Na_2_HPO_4_·12H_2_O) and sodium phosphate monobasic monohydrate (NaH_2_PO_4_·H_2_O) were sourced from Fluka Chemika-BioChemika (Buchs, Switzerland). Sodium dodecyl sulphate (SDS) was obtained from Bio-Rad Laboratories (Hercules, CA, USA). Calcium chloride anhydrous was purchased from QReC™ (Auckland, New Zealand). Bradford protein assay reagent was purchased from Enzmart Biotech Co., Ltd. (Bangkok, Thailand).

### 2.2. Preparation of Printing Inks

SPI was used at a final concentration of 15% (*w*/*v*) in the control formulation, and sodium alginate was added at 0.5% (*w*/*v*). PPHP was incorporated at levels of 0, 2, 4, 6, and 8% (*w*/*w*, based on the total formulation weight) by replacing an equivalent amount of SPI. Distilled water was subsequently added to adjust the formulations to the desired final composition. The mixtures were homogenized using a handheld mixer (Elon Gen 2, Spring Green Evolution, Bangkok, Thailand) for 2 min to ensure uniform dispersion of all components. The resulting suspensions were stored at 4 °C for at least 12 h to facilitate complete hydration of the proteins and PPHP-derived fiber and polysaccharide components. Following hydration, the mixtures were remixed for an additional 1 min to obtain homogeneous printing inks and immediately loaded into 50 mL syringes for subsequent 3D printing.

3D printing was performed using an extrusion-based FoodBot 3D Printer (Changxing Shiyin Technology, Hangzhou, China) at 25 °C. Printing parameters, optimized through preliminary trials, consisted of a 0.84 mm nozzle diameter, printing and retraction speeds of 30 mm/s, 0.84 mm layer height, and a 90% infill density, which together ensured continuous extrusion, adequate layer adhesion, and stable filament deposition with minimal deformation. Cube-shaped constructs (20 × 20 × 5 mm) were designed using CAD software (UltiMaker Cura 15.02.1) and printed to evaluate printability and dimensional stability. Immediately after printing, the constructs were immersed in 2.0% (*w*/*v*) CaCl_2_ for 1 h to induce ionic crosslinking of the alginate matrix and were subsequently heated at 95 °C for 1 h to promote thermal gelation of the SPI-based hydrogel. Samples were cooled to room temperature before further analysis.

### 2.3. Dimensional Printing Deviation and Shape Fidelity

Dimensional accuracy and shape fidelity of the 3D-printed constructs were evaluated against the designed dimensions of the CAD model. Cube-shaped samples (20 × 20 × 2 or 5 mm) were printed under the optimized printing conditions, and their width, length, and height were measured immediately after printing using a digital Vernier caliper (Mitutoyo Corp., Kawasaki, Japan; resolution: 0.01 mm). Each dimension was measured at three different locations per sample, and the mean value was used for subsequent calculations. Three independently printed samples were analyzed for each formulation. The dimensional printing deviation for each dimension (%) was calculated as:

$$Dimensional\;printing\;deviation\;(\%)=\;\frac{\left|D_{m}-D_{d}\right|}{D_{d}}\times 100$$

where *D_m_* is the measured dimension of the printed sample (mm) and *D_d_* is the corresponding designed dimension of the CAD model (mm).

Shape fidelity (%) was calculated by comparing the overall dimensions of the printed construct with those of the designed model:

$$Shape\;fidelity\;\left(\%\right)=\frac{D_{m}}{D_{d}}\times 100$$

Shape fidelity values approaching 100% indicate higher printing accuracy and better structural retention after deposition.

### 2.4. Rheological Measurements

Rheological properties of the SPI–PPHP printing inks were determined using a rotational rheometer (Kinexus Ultra plus, Malvern Instruments Ltd., Malvern, Worcestershire, UK) fitted with a parallel-plate geometry (25 mm diameter, 1.5 mm gap). All measurements were performed at 25 °C unless otherwise specified. Apparent viscosity was measured over a shear rate range of 0.01–100 s^−1^ to evaluate steady-shear flow behavior and shear-thinning characteristics. Strain sweeps (0.1–500%, 10 rad/s) were used to determine the linear viscoelastic region (LVR), from which a strain of 0.2% was selected for subsequent oscillatory tests. Frequency sweeps were performed at 0.2% strain over an angular frequency range of 0.1–100 rad/s, and the storage modulus (*G*′), loss modulus (*G*″), and loss tangent (tan *δ*) were recorded to characterize the viscoelastic properties of the inks. Temperature sweeps were conducted at 0.2% strain and 10 rad/s by heating the samples from 25 to 90 °C and subsequently cooling them from 90 to 25 °C at a constant rate of 5 °C/min. Changes in *G*′ and *G*″ during the heating and cooling cycles were monitored to characterize thermally induced changes in the viscoelastic network and its behavior upon cooling.

### 2.5. Scanning Electron Microscopy (SEM)

The microstructure of the hydrogel samples was examined by scanning electron microscopy (LEO 1450VP, Carl Zeiss, Oberkochen, Germany). Freeze-dried samples were fractured manually to expose internal cross-sections, mounted on aluminum stubs using double-sided conductive carbon tape, and sputter-coated with gold (~10 nm) to enhance conductivity. Micrographs were obtained at an accelerating voltage of 10–15 kV and magnifications of 50× and 100× to evaluate network morphology, including pore structure, compactness, aggregation behavior, and overall microstructural integrity.

### 2.6. Electrophoresis Analysis

Protein profiles of the 3D-printed hydrogels were analyzed by sodium dodecyl sulfate–polyacrylamide gel electrophoresis (SDS–PAGE) according to Laemmli [14] with slight modifications. Briefly, 1.0 g of hydrogel sample was mixed with 9 mL of 10% SDS solution and heated at 95 °C for 1 h, and the dissolved solution was centrifuged (10,000× *g*, 15 min) with the supernatant collected for electrophoretic analysis. Supernatants were mixed (1:1, *v*/*v*) with sample buffer containing 0.5 M Tris–HCl (pH 6.8), 4% (*w*/*v*) SDS, and 20% (*v*/*v*) glycerol, with (reducing) or without (non-reducing) 10% (*v*/*v*) β-mercaptoethanol, and heated in boiling water for 3 min prior to electrophoresis. Protein samples (15 μg per lane) were separated on 12% resolving/4% stacking gels using a Mini-PROTEAN^®^ Tetra Cell system (Bio-Rad Laboratories, Hercules, CA, USA) at a constant current of 15 mA per gel for 110 min, 120 V. Gels were stained with 0.1% (*w*/*v*) Coomassie Brilliant Blue R-250 (15% *v*/*v* methanol, 5% *v*/*v* acetic acid) and destained (30% *v*/*v* methanol, 10% *v*/*v* acetic acid) until clear bands appeared, with molecular weight standards (10–250 kDa) used to estimate protein molecular masses.

### 2.7. Texture Profile Analysis (TPA)

Textural properties of the 3D-printed hydrogels were determined by TPA using a TA.XTplus Texture Analyzer (Stable Micro Systems Ltd., Godalming, UK) fitted with a 35 mm cylindrical probe (P/35) and a 1 kg load cell. Cylindrical samples (25 mm diameter × 20 mm height) were equilibrated at 25 ± 2 °C prior to analysis and compressed twice to 50% of their original height (5 s interval between cycles) at a trigger force of 5 g and a pre-test, test, and post-test speed of 2 mm/s. Hardness, adhesiveness, springiness, cohesiveness, gumminess, and chewiness were calculated automatically from the force–time curves. Measurements were performed in ten replicates.

### 2.8. Fourier-Transform Infrared (FTIR) Spectroscopy

FTIR spectroscopy was used to evaluate protein secondary structure according to the method described previously [15], with minor modifications. Spectra were acquired using a Bruker Tensor 27 spectrometer equipped with a DTGS detector and diamond ATR accessory. Each spectrum was collected from 4000 to 400 cm^−1^ at 4 cm^−1^ resolution using 64 scans. Spectra were processed using Unscrambler X 10.5, and second derivatives were calculated using the Savitzky–Golay algorithm. Principal component analysis (PCA) was subsequently performed to evaluate spectral variation among samples. For secondary structure analysis, the amide I region (1600–1700 cm^−1^) was baseline-corrected and subjected to Fourier self-deconvolution. Peak positions were identified from second-derivative spectra and fitted in PeakFit 4.12 using Gaussian functions. Relative secondary structure contents were calculated from the integrated areas of the fitted bands assigned to α-helix, β-sheet, β-turn, and antiparallel β-sheet. Fits with correlation coefficient (*r*^2^) > 0.95 and reduced chi-square (*χ*^2^) < 0.001 were accepted.

### 2.9. In Vitro Gastrointestinal Digestion (IVPD)

*In vitro* gastrointestinal digestion was performed according to the standardized INFOGEST static digestion protocol with minor modifications [16,17]. The oral digestion phase was omitted, as the objective of this study was to investigate protein digestion, whereas the gastric and intestinal digestion procedures were conducted following the INFOGEST protocol. For the digestion experiment, three formulations (Control, PPHP4, and PPHP8) were selected to represent the formulation without PPHP, an intermediate PPHP level, and the highest PPHP level, respectively. These formulations were selected to provide a representative comparison across the PPHP concentration range while limiting the number of digestion samples for the detailed time-course analysis. Prior to digestion, 1.0 g of each 3D-printed hydrogel sample was mixed with 20 mL of distilled water and homogenized using a homogenizer (HG-15D, Daihan Scientific, Wonju-si, Republic of Korea) at 8000 rpm for 1 min to obtain a uniform sample dispersion. For the gastric phase, 10 mL of protein solution (50 mg/mL) was combined with 9 mL of simulated gastric fluid (SGF: consisted of 6.9 mmol/L KCl, 0.9 mmol/L KH_2_PO_4_, 25 mmol/L NaHCO_3_, 47.2 mmol/L NaCl, 0.5 mmol/L (NH_4_)_2_CO_3_ and 0.12 mmol/L MgCl_2_·6H_2_O) and 10 μL of 0.3 mol/L CaCl_2_. The pH was adjusted to 3.0 using 6 N HCl prior to the addition of pepsin to achieve a final enzyme activity of 2000 U/mL. The reaction volume was adjusted to 20 mL with distilled water and incubated at 37 °C for 2 h. At the end of gastric digestion, an aliquot was collected for subsequent analyses. To initiate the intestinal phase, 20 mL of simulated intestinal fluid (SIF: consisted of 6.8 mmol/L KCl, 0.8 mmol/L KH_2_PO_4_, 85 mmol/L NaHCO_3_, 38.4 mmol/L NaCl, and 0.33 mmol/L MgCl_2_·6H_2_O) and 80 μL of 0.3 mol/L CaCl_2_ were added to the gastric digesta. The pH was then adjusted to 7.0 using 0.1 N NaOH. Pancreatin was incorporated to obtain a final enzyme activity of 100 U/mL, and the total reaction volume was adjusted to 40 mL with distilled water. Bile salts are added to achieve a final concentration of 10 mM in the final mixture. The digestion mixture was further incubated at 37 °C for 2 h. Aliquots (200 μL) were collected at 30 min intervals throughout the digestion process for subsequent analyses. At the end of the intestinal digestion, enzymatic activity was terminated by heating the remaining digestion mixture in a boiling water bath for 5 min. The digesta were subsequently centrifuged, and the resulting supernatants were collected and stored as the gastrointestinally digested samples for degree of hydrolysis (DH) analyses.

The DH was determined using the trinitrobenzenesulfonic acid (TNBS) assay [18], which quantifies the free amino groups released during enzymatic hydrolysis of peptide bonds. Briefly, the digested samples were reacted with TNBS reagent, and the absorbance was measured at 340 nm. L-Leucine was used as the calibration standard to construct the standard curve, and the concentration of free amino groups was expressed as L-leucine equivalents. The DH was calculated from the increase in free amino groups relative to the total number of peptide bonds in the protein. *In vitro* protein digestibility was calculated based on the amount of free amino acids released after simulated gastrointestinal digestion.

### 2.10. In Vitro Prebiotic Effects Analysis

#### 2.10.1. Bacterial Preparation

*Bifidobacterium longum* subsp. *longum* TBRC 7151 was cultured in Gifu anaerobic broth (GAM; Himedia, Thane, India) adjusted to pH 7.4 and incubated at 37 °C for 48 h under anaerobic condition. GAM was used as supplied by the manufacturer, and no additional carbon source, such as fructooligosaccharides (FOS) or inulin, was added. Bacterial growth was monitored by measuring the optical density at 600 nm (OD_600_) using a UV–Vis spectrophotometer (Thermo Fisher Scientific, Waltham, MA, USA). The bacterial culture reached an OD_600_ value of 1.00 ± 0.05 before use in the subsequent experiment. Viable bacterial counts were determined using the standard plate-count method on GAM agar following incubation at 37 °C under anaerobic conditions.

#### 2.10.2. Effect of PPHP on the Growth of *B. longum* subsp. *longum*

Five formulations were prepared using a total of 8 g of solid ingredients, consisting of SPI, PPHP, and alginate. SPI was partially replaced with PPHP at increasing levels. The control formulation contained 7.5 g of SPI without peach palm. For treatments PPHP2, PPHP4, PPHP6, and PPHP8, the amount of SPI was reduced to 6.5, 5.5, 4.5, and 3.5 g, respectively, while peach palm was added at 1, 2, 3, and 4 g, respectively. Alginate was maintained at 0.5% (*w*/*w*) in all formulations. Distilled water (42 mL) was added to each formulation, and the mixtures were thoroughly homogenized and incubated at 4 °C for 6–12 h before being sterilized by autoclaving at 121 °C for 15 min. After sterilization, the formulations were allowed to cool to room temperature. The *B. longum* subsp. *longum* suspension was added into each formulation and thoroughly mixed to achieve a final bacterial concentration of 1 × 10^3^ CFU/mL. In addition, GAM broth inoculated with *B. longum* subsp. *longum* at the same final bacterial concentration (1 × 10^3^ CFU/mL) was included as the culture medium control (C-GAM). All formulations and the culture-medium control were incubated at 37 °C for 48 h under anaerobic conditions. Following incubation, viable cell counts of *B. longum* subsp. *longum* were determined using the standard plate-count method. Serial 10-fold dilutions of each sample were prepared in phosphate-buffered saline (PBS; pH 7.4), and appropriate dilutions were spread onto GAM agar plates. The plates were subsequently incubated at 37 °C for 48 h under anaerobic conditions.

### 2.11. Statistical Analysis

Experiments were conducted using at least three independent replicates, with the exact number of replicates varying among analytical measurements according to the experimental procedure. The number of replicates (*n*) for each analysis is reported in the corresponding tables and figure captions. Results are expressed as mean ± standard deviation (SD). Texture profile analysis (TPA) was performed using 10 independent measurements per formulation (*n* = 10). Statistical analyses were performed using Minitab^®^18 Statistical Software (Minitab LLC, State College, PA, USA). Differences among treatments were analyzed by one-way analysis of variance (ANOVA) followed by Tukey’s honestly significant difference (HSD) test. For the in vitro digestion study, the effects of treatment and digestion time were evaluated using two-way ANOVA. Statistical significance was established at *p* < 0.05.

## 3. Results and Discussion

### 3.1. Rheological Properties of SPI-PPHP Inks

**Figure 1:**
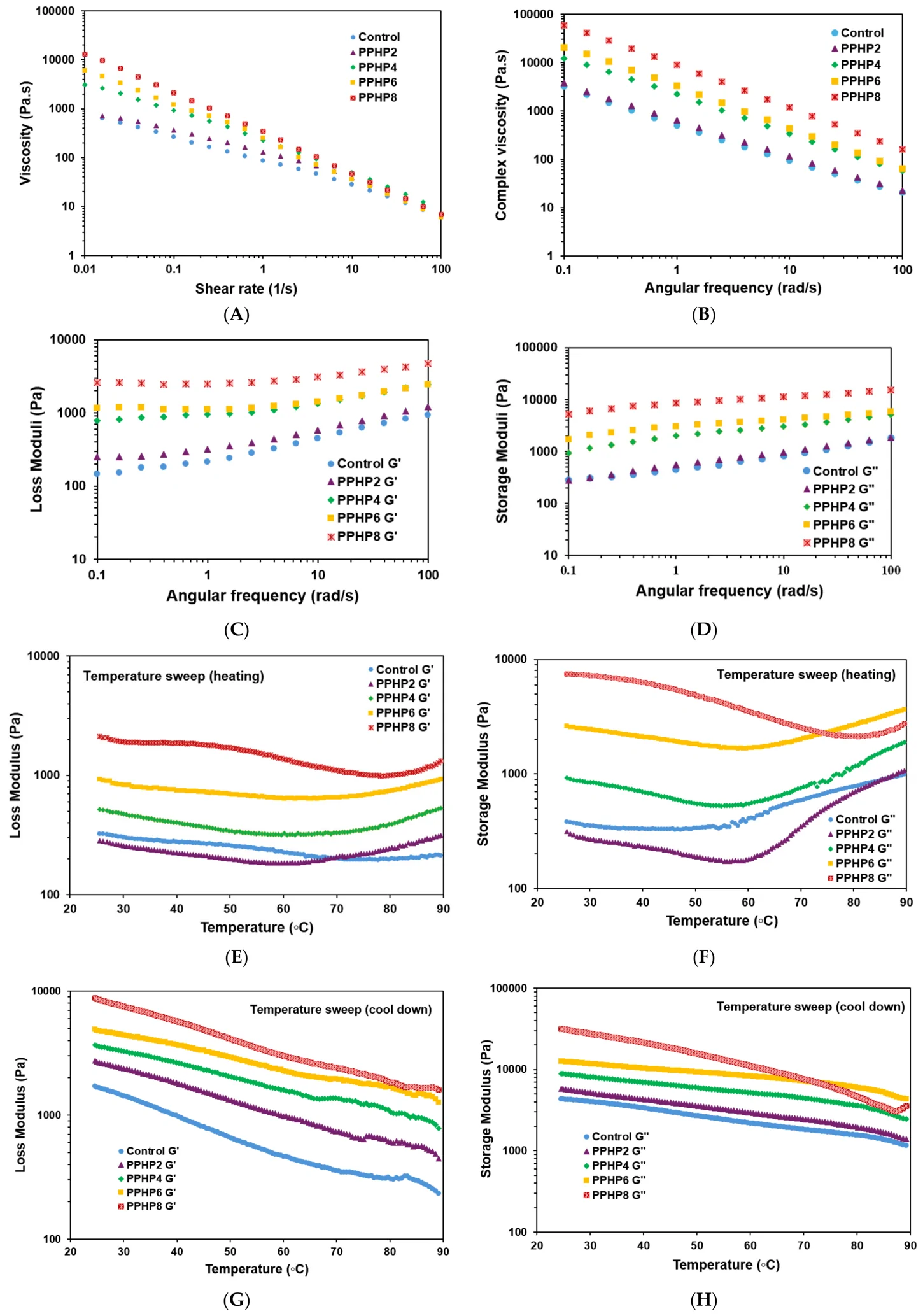
Rheological properties of SPI–PPHP inks. Apparent viscosity as a function of shear rate (**A**); complex viscosity as a function of angular frequency (**B**); loss modulus (*G*″) (**C**) and storage modulus (*G*′) (**D**) as a function of angular frequency; loss modulus (*G*″) and storage modulus (*G*′) (**E**,**F**), respectively, during heating; and loss modulus (*G*″) and storage modulus (*G*′) (**G**,**H**), respectively, during cooling. C, control SPI; PPHP2–PPHP8, SPI formulations containing increasing concentrations of PPHP.

The rheological behavior of the SPI–PPHP formulations demonstrated a concentration-dependent increase in viscosity and viscoelastic strength with increasing PPHP concentration (Figure 1). Under steady shear conditions, all formulations exhibited pronounced shear-thinning behavior, with viscosity decreasing continuously as the shear rate increased from 0.01 to 100 s^−1^ (Figure 1A). The control showed the lowest viscosity over most of the shear-rate range, whereas incorporation of PPHP progressively increased the viscosity, with PPHP8 exhibiting the highest values at low and intermediate shear rates. These results indicate that increasing PPHP incorporation, together with the corresponding reduction in SPI concentration, modified the flow resistance and structural organization of the formulations. The pronounced shear-thinning behavior is particularly advantageous for extrusion-based 3D printing because the ink can maintain sufficient viscosity and structural integrity at rest while becoming less viscous under the high shear conditions encountered during extrusion, facilitating material flow through the nozzle [19,20]. The increase in viscosity with PPHP concentration may be associated with greater water immobilization and increased intermolecular association within the SPI–PPHP matrix. The dietary fiber and polysaccharide components of PPHP can increase the effective hydrodynamic volume of the dispersed phase and promote physical interactions with SPI, thereby increasing resistance to flow. At low shear rates, these interactions may contribute to the formation of a more structured matrix, resulting in higher apparent viscosity [21]. As shear rate increases, alignment and rearrangement of protein and PPHP-derived macromolecular components progressively reduce resistance to flow, accounting for the observed shear-thinning response [22].

The frequency sweep measurements further demonstrated that PPHP substantially enhanced the viscoelastic properties of the inks (Figure 1B–D). The complex viscosity decreased with increasing angular frequency, indicating frequency-dependent disruption or rearrangement of the ink structure under oscillatory deformation. However, the magnitude of complex viscosity increased systematically with PPHP concentration, with PPHP8 showing the highest values throughout the tested frequency range. This indicates that PPHP increased the resistance of the formulations to oscillatory deformation and promoted a more structured viscoelastic matrix. The *G*′ and *G*″ profiles provided further evidence of the structural reinforcement induced by PPHP. For all formulations, *G*′ remained higher than *G*″ throughout the frequency range, indicating a predominantly elastic or gel-like character. Importantly, both *G*′ and *G*″ increased with increasing PPHP concentration, with PPHP8 displaying the highest moduli. The greater increase in *G*′ compared with *G*″ suggests that PPHP primarily strengthened the elastic component of the matrix. A high *G*′ is desirable for extrusion-based food printing because the deposited material must rapidly resist deformation after leaving the nozzle and support subsequent layers [23]. The stronger elastic network in PPHP-containing inks may therefore contribute to improved post-deposition shape retention and reduced dimensional spreading.

The temperature-sweep results revealed that the viscoelastic properties of all formulations were temperature-dependent (Figure 1E–H). During heating, *G*′ generally decreased initially and reached a minimum before increasing at higher temperatures, whereas *G*″ showed a similar temperature-dependent transition. The reduction in moduli during the initial heating stage likely reflects thermally induced weakening and rearrangement of non-covalent interactions within the hydrated SPI–PPHP network [24]. At higher temperatures, the subsequent increase in moduli may be associated with thermally induced protein aggregation and network restructuring, which increase the rigidity of the system [25]. These changes were particularly pronounced for the PPHP-containing formulations, with PPHP8 maintaining substantially higher *G*′ and *G*″ values than the control throughout heating. During cooling, the *G*′ and *G*″ profiles changed progressively as the temperature decreased from 90 to 25 °C, with the PPHP-containing formulations maintaining higher moduli than the control throughout most of the cooling cycle (Figure 1G,H). The relatively high moduli after thermal treatment suggest that PPHP-containing matrices maintained greater viscoelastic strength following the heating–cooling cycle. PPHP8 exhibited the highest viscoelastic moduli during most of the cooling process, indicating the strongest post-heating viscoelastic network among the formulations evaluated. However, the SPI–PPHP system is compositionally complex, and the observed temperature-dependent changes may also involve other concurrent mechanisms, such as polysaccharide gelation or association, competition for water between protein and polysaccharide components, and changes in hydration and molecular mobility.

**Figure 2:**
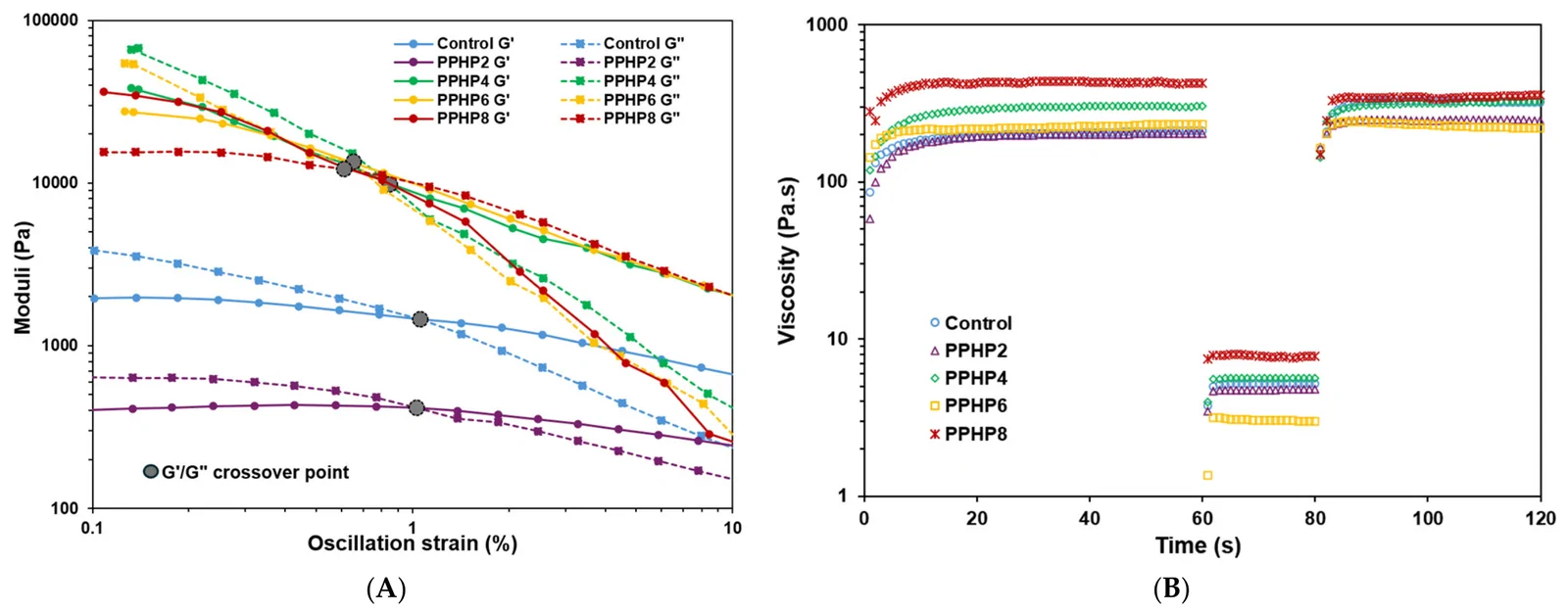
Oscillatory rheology (**A**) and structural recovery (**B**) of SPI-PPHP inks.

The oscillatory rheological behavior and structural recovery of the control and PPHP-containing inks are shown in Figure 2. In strain sweep tests (Figure 2A), all formulations exhibited predominantly elastic behavior (*G*′ > *G*″) at low strain, indicating a structured network capable of resisting deformation within the LVR. PPHP incorporation increased both *G*′ and *G*″, with the highest moduli observed for PPHP8, suggesting greater matrix association and resistance to deformation. Similar concentration-dependent increases in modulus have been reported for SPI-based inks, reflecting greater resistance to deformation but potentially higher extrusion stress [22]. As strain increased, *G*′ progressively decreased and *G*″ increased, indicating network disruption and a transition toward flow, consistent with the strain-dependent structural breakdown reported by Ji et al. [26]. The *G*′–*G*″ crossover at approximately 1% strain marked the transition from predominantly elastic to viscous behavior. The shift in crossover behavior observed for PPHP-containing inks suggests increased resistance to strain-induced structural breakdown, while their higher moduli indicate greater structural robustness prior to extrusion.

Time-dependent viscosity measurements (Figure 2B) further demonstrated the structural recovery behavior of the inks following high-shear deformation. During the initial low-shear interval, all inks rapidly reached a stable viscosity plateau that increased with PPHP concentration (PPHP8 > PPHP6 > PPHP4 > PPHP2 > control), consistent with the concentration-dependent rheological behavior observed in the preceding tests. Under high shear, viscosity decreased markedly for all samples, confirming shear-thinning behavior favorable for extrusion. Upon shear removal, viscosity recovered rapidly toward the pre-shear level, demonstrating substantial structural reformation. The higher recovered viscosities of PPHP-containing inks suggest that PPHP promoted greater post-shear structural rebuilding, although direct comparison of absolute recovered viscosity alone does not necessarily indicate a higher percentage recovery relative to the initial value. The observed shear-thinning and rapid viscosity recovery of the PPHP-containing inks were consistent with the findings of Guo et al. [27], who reported that these properties facilitate extrusion and subsequent structural recovery after printing. Collectively, PPHP incorporation increased viscosity, viscoelastic strength, resistance to deformation, and post-shear structural rebuilding in SPI-based inks. These concentration-dependent rheological changes provide a plausible physicochemical basis for the improved dimensional accuracy and shape retention of the PPHP-containing printed constructs.

The rheological results collectively demonstrate that PPHP incorporation produced a more viscous and predominantly elastic SPI-based printing matrix while preserving desirable shear-thinning behavior. Increasing PPHP concentration enhanced low-shear viscosity, complex viscosity, *G*′, and *G*″, with PPHP8 exhibiting the strongest rheological response among the formulations tested. These characteristics indicate greater resistance to deformation before and after extrusion and are consistent with the improved dimensional accuracy and shape retention observed in PPHP-containing printed samples. Among the concentrations evaluated, PPHP8 provided the most favorable rheological characteristics for balancing extrusion flow and post-deposition structural stability under the printing conditions employed in this study.

### 3.2. Printability and Shape Fidelity of 3D-Printed Samples

PPHP incorporation markedly improved the dimensional accuracy and shape fidelity of 3D-printed samples (Table 1). The control showed the largest deviations (width 4.25%, height 7.00%; overall 5.63%), while increasing PPHP concentration progressively reduced these values, reaching only 0.25% for both dimensions in PPHP8. This trend indicates that PPHP enhanced the ink’s ability to retain its programmed geometry during and after printing. This improvement likely stems from PPHP’s polysaccharide components, which increase ink consistency and viscoelasticity, thereby improving resistance to post-extrusion deformation. A stronger internal network can rapidly support deposited layers, minimizing lateral spreading and gravitational sagging before stabilization. The effect was most pronounced in height retention, where deviations dropped from 7.00% (control) to 0.83% (PPHP6) and 0.25% (PPHP8), reflecting improved structural support across successive layers.

Shape fidelity values corroborated these findings. The control exhibited 104.25% shape fidelity, indicating notable spreading beyond the designed dimensions, whereas fidelity progressively approached the ideal 100% with increasing PPHP concentration (103.50%, 101.92%, 101.17%, and 100.25% for PPHP2–PPHP8, respectively). PPHP8 thus achieved near-perfect dimensional matching, confirming that PPHP effectively suppressed post-extrusion spreading. These results suggest that PPHP concentration critically governs printability in SPI-based inks. Although the 0.25% dimensional deviation observed for PPHP8 indicates highly accurate reproduction of the designed geometry, this finding should be interpreted within the scope of the present study, as the printed constructs were relatively small and geometrically simple (20 × 20 × 5 mm). Similar accuracy may not hold for larger, multilayered, or more complex structures, where gravitational deformation, cumulative layer-to-layer errors, and prolonged extrusion could affect shape retention. Further studies using larger, more complex geometries are needed to confirm the scalability of this printing accuracy. At lower concentrations, the polysaccharide network may be insufficient to resist deformation, leading to greater spreading. Liu et al. [22] demonstrated that increasing ink concentration improved shape fidelity and enabled the printing of more layers while maintaining structural integrity. As PPHP concentration increases, stronger polymer–protein and polysaccharide–polysaccharide interactions likely produce a more interconnected matrix capable of withstanding its own weight and printing-induced stresses. However, excessive matrix strengthening could impair extrudability, suggesting that 8% PPHP represents an optimal balance between flow and post-deposition stability under the tested conditions.

**Figure 3:**
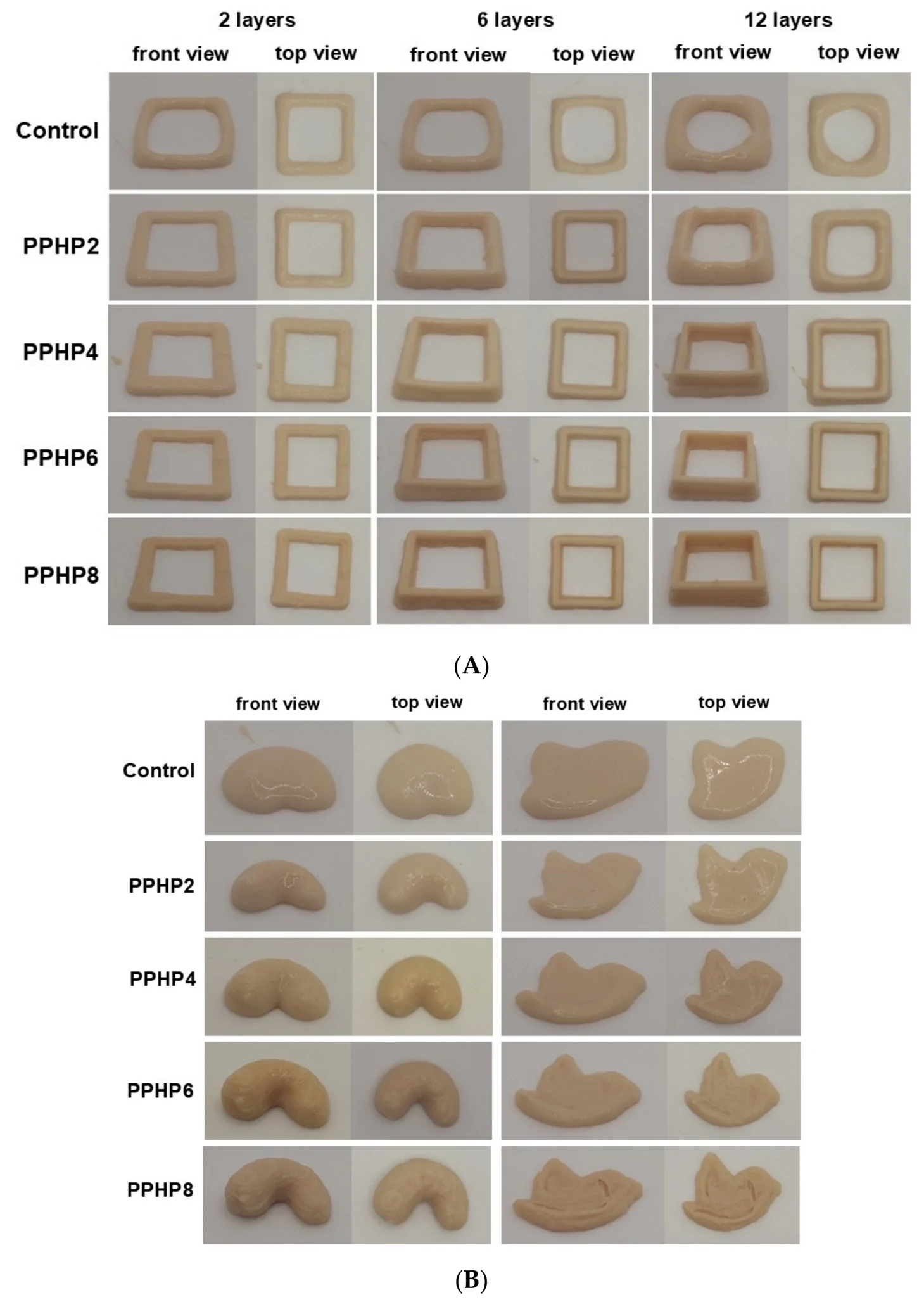
Representative images demonstrating the effect of PPHP incorporation on the printability and shape fidelity of 3D-printed samples: square-shaped samples (**A**) and samples with various geometries (**B**).

Representative images of the printed samples (Figure 3A,B) revealed clear differences in printability and shape fidelity among formulations. The control retained the overall designed geometry but showed noticeable dimensional distortion, especially at edges and corners, reflecting limited post-deposition structural stability. In contrast, samples containing functional ingredients displayed improved filament deposition with sharper edges and more uniform structures, consistent with their enhanced shear-thinning behavior and elastic character, which promote smooth extrusion and rapid structural recovery after deposition. These visual observations align with the dimensional analysis, where formulations exhibiting greater rheological stability also showed lower printing deviation and higher shape fidelity. A stronger viscoelastic network resists gravitational spreading and layer deformation, preserving the printed geometry. Together, these results confirm that the modified SPI formulations achieved improved printability and post-deposition structural stability. The progressive reduction in dimensional deviation and convergence of shape fidelity toward 100% confirm that PPHP substantially enhanced the geometric accuracy of SPI-based printed samples, with PPHP8 (0.25% deviation, 100.25% fidelity) demonstrating strong potential as a polysaccharide-based structuring agent for protein-based 3D-printed foods.

### 3.3. Microstructural Characteristics

**Figure 4:**
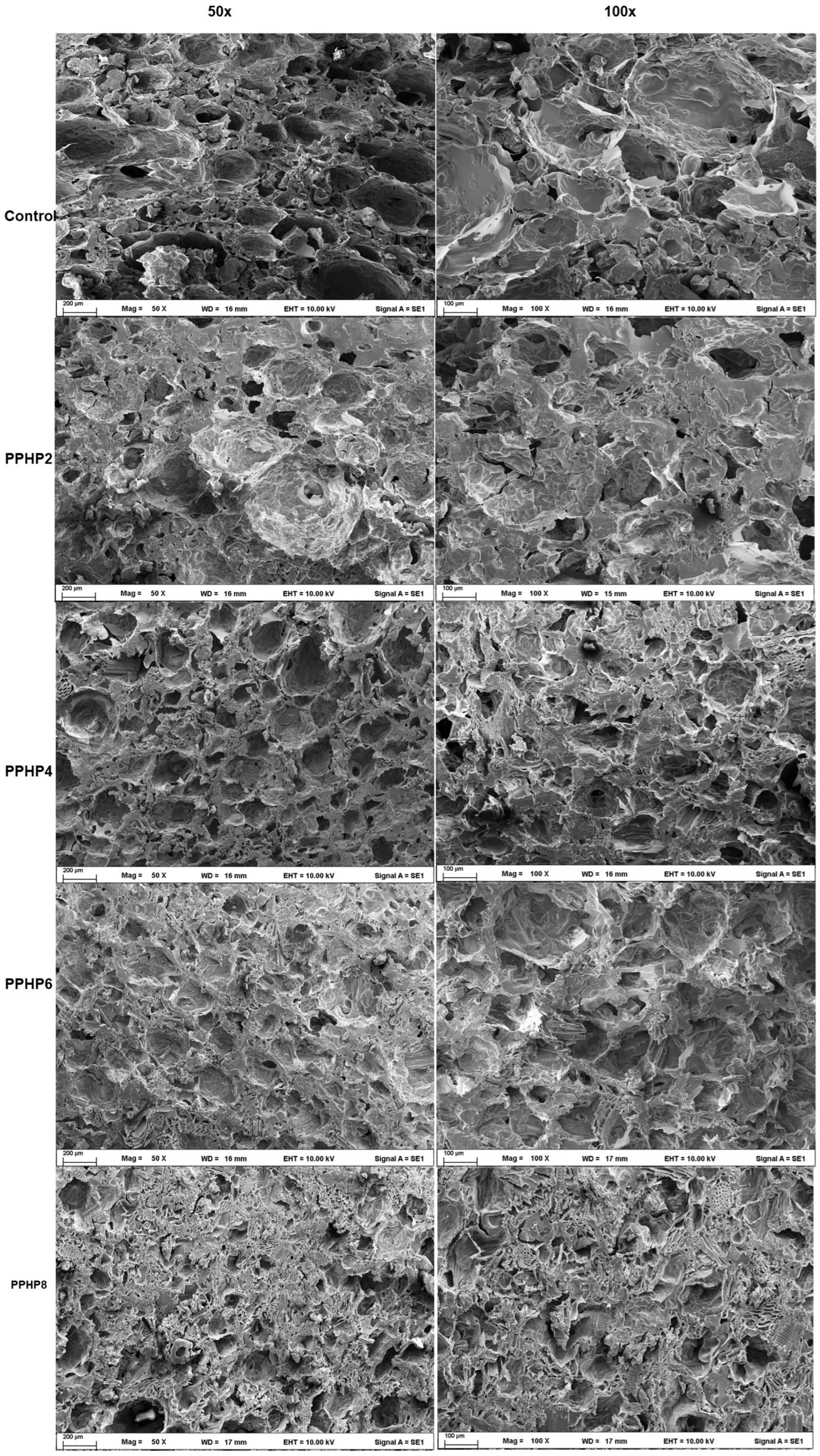
Microstructure of SPI–PPHP gels at different PPHP concentrations at 50× and 100× magnification.

The microstructural characteristics of SPI-based gels containing different PPHP concentrations are shown in Figure 4. The control gel displayed a continuous, interconnected protein network, formed through rearrangement and aggregation of unfolded soy proteins during thermal treatment, resulting in a porous matrix capable of entrapping water—an essential feature for maintaining structural integrity and printability during extrusion. With increasing PPHP concentration, polysaccharide fibers became progressively distributed throughout the SPI matrix, producing a more heterogeneous microstructure. This suggests that PPHP functioned as a structural filler, likely interacting with soy proteins via hydrogen bonding and other non-covalent interactions between polysaccharide hydroxyl groups and protein functional groups, thereby reinforcing and stabilizing the gel network [28]. At higher PPHP concentrations, micrographs showed a qualitative shift in gel matrix organization, with more visible PPHP-derived fibrous material distributed within the protein network, suggesting increased structural heterogeneity and protein–fiber interaction. However, as SEM was used only for qualitative comparison without quantitative pore-size or porosity analysis, the formation of a denser protein–polysaccharide network cannot be conclusively established from these images alone. These microstructural changes are therefore interpreted as qualitative evidence potentially contributing to the improved rheological stability and shape fidelity observed at higher PPHP concentrations. This reflects a trade-off between fiber-mediated reinforcement and protein network dilution. PPHP successfully integrated into the SPI gel network, forming a composite protein–polysaccharide structure that likely underlies the enhanced viscoelastic properties and shape fidelity of the 3D-printed samples.

### 3.4. Electrophoresis Analysis

**Figure 5:**
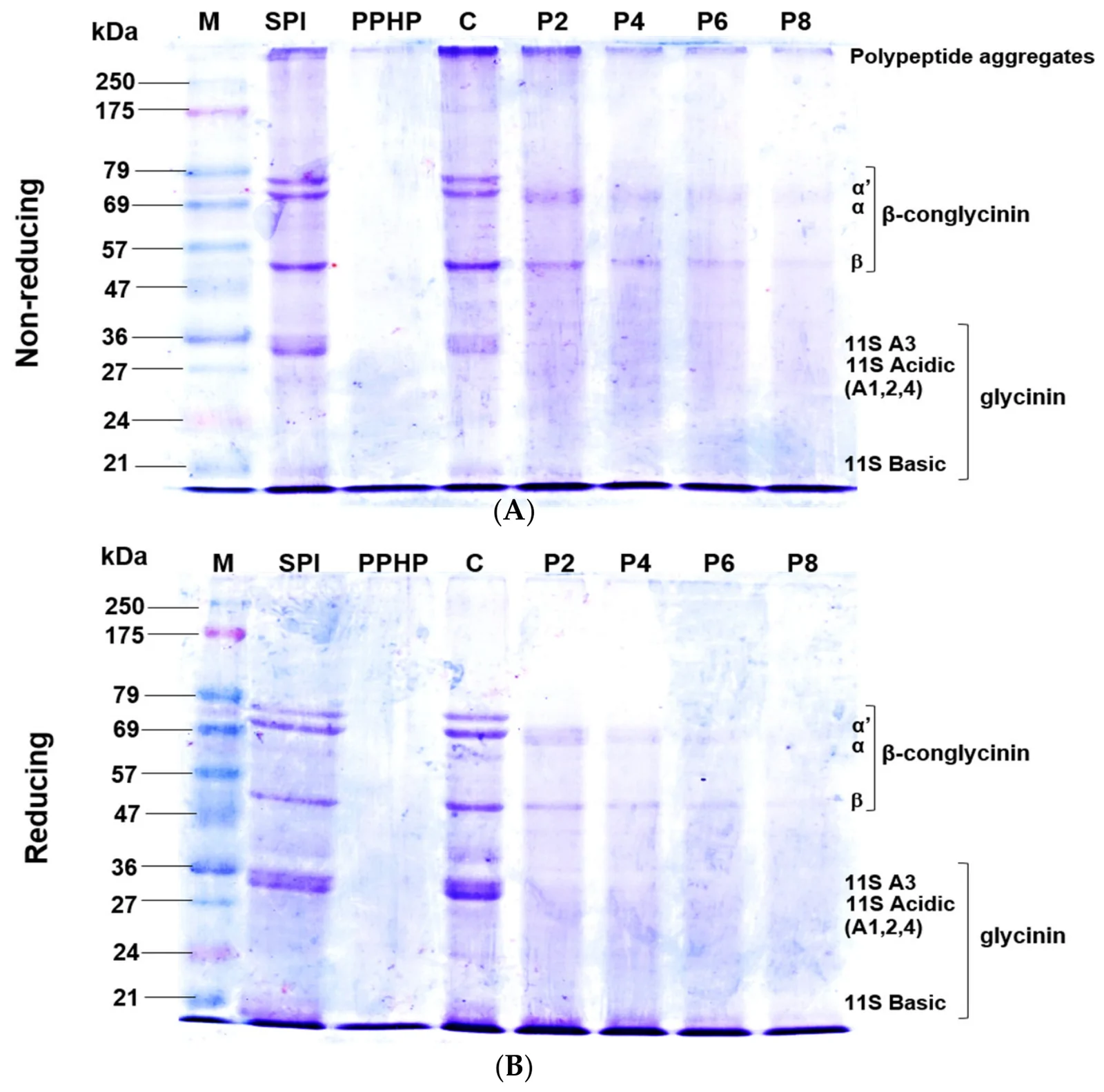
SDS–PAGE profiles of SPI gels containing different concentrations of PPHP under non-reducing (**A**) and reducing conditions (**B**). M, molecular weight marker; C, control SPI gel; P2–P8, SPI gels containing increasing concentrations of PPHP.

To elucidate the molecular mechanisms underlying the rheological and printability improvements described above, SDS–PAGE was performed under non-reducing and reducing conditions (Figure 5). The control SPI gel (C) displayed characteristic soybean storage protein bands—β-conglycinin subunits (α′, α, β; ~47–75 kDa) and glycinin subunits (acidic, ~30–40 kDa; basic, ~20 kDa)—along with a high-molecular-weight fraction (>175 kDa) reflecting aggregates stabilized by disulfide bonds and non-covalent interactions during gelation. With increasing PPHP concentration, intensity of the major SPI bands decreased progressively under non-reducing conditions, while high-molecular-weight material accumulated near the stacking region. PPHP-derived polysaccharides likely engage soy proteins through hydrogen bonding, hydrophobic association, and physical entrapment, sequestering a fraction of protein into complexes too large to migrate freely—consistent with similar behavior reported in other plant protein–polysaccharide systems [29,30]. Since disulfide cleavage would be expected to reduce disulfide-mediated aggregation, the persistence of attenuated β-conglycinin and glycinin bands with residual high-molecular-weight material under reducing conditions is consistent with interactions other than disulfide bonds, including non-covalent associations. However, SDS–PAGE alone cannot conclusively distinguish non-covalent interactions from other covalent cross-links that may not produce readily detectable changes in electrophoretic mobility [31]. These findings are consistent with physically entangled or adsorptively associated protein–polysaccharide complexes, rather than direct evidence of PPHP-promoted covalent cross-linking. More sensitive analyses, such as free thiol determination or size-exclusion chromatography, would be needed to directly assess the presence and extent of covalent cross-linking. These molecular observations provide a mechanistic basis for the macroscopic trends reported earlier. Restricted protein mobility and formation of an SDS-resistant composite network are consistent with PPHP acting as a structural filler that reinforces the SPI matrix while limiting excessive protein aggregation. This directly rationalizes the elevated viscosity and storage modulus observed rheologically, as well as the reduced dimensional deviation and near-ideal shape fidelity at higher PPHP concentrations, linking specific protein–polysaccharide interactions to printing performance. Collectively, these findings support a model in which PPHP actively remodels the protein matrix—forming a composite network stabilized by both non-covalent and, to a lesser extent, covalent interactions—rather than simply diluting it. This dual-mode association likely explains the balance between enhanced structural integrity and the moderate softening observed in texture profile analysis at excessive PPHP levels, as protein–polysaccharide complexation competes with native protein aggregation.

### 3.5. TPA

TPA of the 3D-printed SPI samples showed formulation-dependent numerical changes in several textural parameters following PPHP incorporation, although not all differences were statistically significant (Table 2). The control exhibited the highest hardness (2473.01 g), which decreased markedly at 4–8% PPHP, suggesting that PPHP disrupted formation of a compact protein network. The reduction in hardness with increasing PPHP incorporation may be associated with both the presence of PPHP-derived polysaccharides/fiber and the concomitant reduction in SPI concentration. The lower protein concentration may reduce the density of the protein network formed during gelation, while the incorporation of hydrated fiber and polysaccharide components may further modify the matrix structure and mechanical response. Therefore, the present experimental design does not allow the contribution of PPHP-specific effects to be distinguished from protein dilution. Similarly, Jin et al. [32] reported that cellulose incorporation may disrupt protein network connectivity. Gumminess and chewiness followed the same trend, with the control showing the highest values (1196.68 g and 796.19 g·mm, respectively) and both parameters declining with increasing PPHP concentration, consistent with reduced hardness and cohesiveness. This weaker internal bonding may result from insoluble fiber particles acting as discontinuous phases that limit continuous protein network formation—an effect reported previously in fiber-enriched plant protein gels, where improved water retention often comes at the expense of network compactness and firmness [33,34]. Adhesiveness became more negative with increasing PPHP concentration, with PPHP6 and PPHP8 significantly higher than the control, likely due to enhanced surface moisture retention from hydrated fiber components increasing probe–sample interaction during compression. Springiness remained unaffected (0.641–0.676 mm), indicating that elastic recovery was preserved, possibly through intermolecular SPI–alginate interactions that maintained structural flexibility [35]. Although PPHP reduced firmness and mechanical strength, this may be advantageous for plant-based meat analogues, where excessive hardness is undesirable. The softer texture, increased adhesiveness, and reduced chewiness suggest that PPHP can shift SPI-based printed products toward a more meat-like textural profile. Thus, PPHP incorporation reduced hardness, cohesiveness, gumminess, chewiness, and resilience while preserving springiness. Combined with the improved printability and dimensional stability described earlier, these results highlight PPHP’s potential as a functional ingredient for tailoring the texture of 3D-printed protein foods.

### 3.6. Molecular Secondary Structure by FTIR

**Figure 6:**
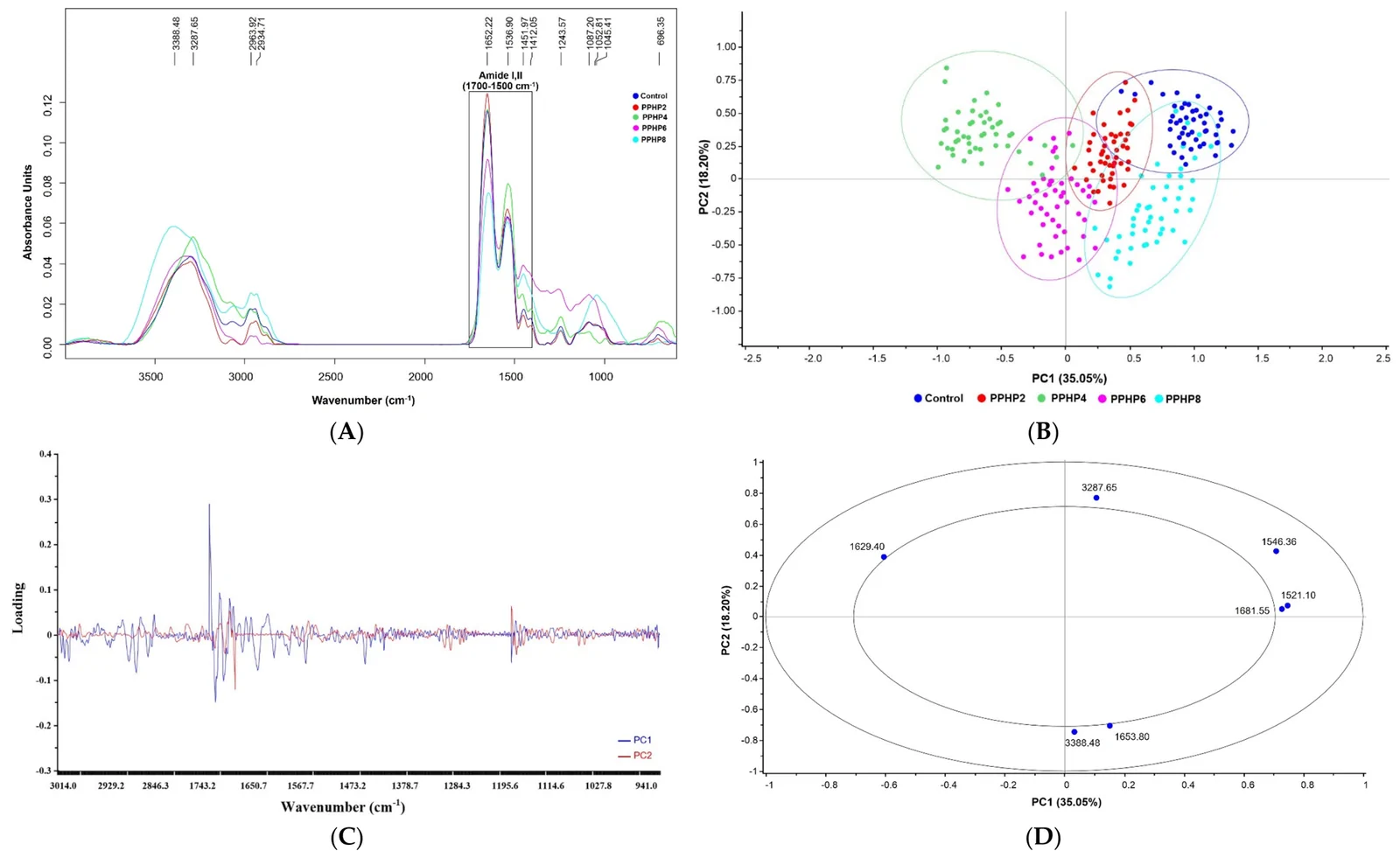
Average original FTIR spectra (**A**), PCA score plot (**B**), loading plot (**C**), and correlation loading plot (**D**) of SPI-PPHP gels at different PPHP concentrations. The obtained spectra were analyzed in the regions of 3000–2800 cm^−1^ and 1800–900 cm^−1^.

The original FTIR spectra of SPI–PPHP gels revealed characteristic vibrational regions at 3200–3500, 2850–2950, 1600–1700, 1500–1600, and 1200–1000 cm^−1^ (Figure 6A), corresponding mainly to O–H/N–H stretching, aliphatic C–H stretching, amide I, amide II, and C–O/C–O–C stretching vibrations, respectively [36]. The band at 3200–3500 cm^−1^ became broader with increasing PPHP concentration, suggesting enhanced hydrogen-bonding interactions between PPHP and SPI. Shifts in the 2850–2950 cm^−1^ bands, associated with CH_2_ and CH_3_ stretching, may further reflect changes in the local molecular environment resulting from SPI–PPHP interactions. The amide I (1600–1700 cm^−1^) and amide II (1500–1600 cm^−1^) regions were predominant across all samples, reflecting the protein-rich nature of the gel matrix. Meanwhile, changes in the 1200–1000 cm^−1^ region, associated with C–O and C–O–C stretching, may reflect contributions from carbohydrate-rich components of PPHP and alterations in their molecular environment upon incorporation into the SPI matrix [37]. Overall, PPHP incorporation did not introduce major new functional groups but altered band intensity and position, suggesting intermolecular interactions between SPI and PPHP, particularly through hydrogen bonding and other non-covalent interactions.

The major biomolecular regions (3000–2800 and 1800–900 cm^−1^) were further subjected to PCA. The score plot (Figure 6B) explained 53.25% of the total variance, with PC1 and PC2 accounting for 35.05% and 18.20%, respectively. PC1 mainly separated PPHP4 and PPHP6 from the Control, PPHP2, and PPHP8, while PC2 further differentiated PPHP6 and PPHP8 from the Control and PPHP4. Despite partial overlap, the distinct clustering suggested PPHP-induced changes in the molecular environment of the SPI gel matrix, consistent with the spectral shifts and broadening observed in the original spectra. The loading and correlation loading plots (Figure 6C,D) identified seven discriminating bands at 1521.10, 1546.36, 1629.40, 1653.80, 1681.55, 3287.65, and 3388.48 cm^−1^ (*p* < 0.05). The band at 1629.40 cm^−1^ on negative PC1 was associated with PPHP4 and PPHP6, whereas those at 1521.10, 1546.36, and 1681.55 cm^−1^ were more closely associated with the Control, PPHP2, and PPHP8. Along PC2, the 3388.48 and 1653.80 cm^−1^ bands were associated with PPHP6 and PPHP8, contributing to their differentiation from the Control. These discriminating bands, mainly located within the O–H/N–H stretching and amide I/II regions, suggest that PPHP incorporation altered hydrogen-bonding interactions and protein conformation within the gel matrix [18]. The prominent contribution of the amide I bands further indicated that PPHP-induced spectral differentiation was closely associated with changes in protein secondary structure, which was subsequently examined by deconvolution of the amide I region.

The relative contents of protein secondary structures, determined by second-derivative analysis and curve fitting of the amide I region (Figure S1), are presented in Table 3. Control exhibited the highest α-helix content (36.94%), which decreased significantly following PPHP incorporation, reaching the lowest value in PPHP4 (28.10%) (*p* < 0.05). In contrast, β-sheet content increased from 26.45% in the Control to 30.33–32.04% in PPHP-containing gels, with the highest value observed in PPHP4 (*p* < 0.05). Antiparallel structures showed a similar increase, reaching a maximum of 21.45% in PPHP4, whereas β-turn content remained relatively unchanged (18.41–19.72%) (*p* > 0.05). These changes indicate that PPHP promoted a transition from the more ordered α-helical conformation toward β-sheet-rich structures, reflecting unfolding and subsequent rearrangement of SPI during formation of the protein–polysaccharide gel network. The PPHP4 formulation exhibited the lowest α-helix content and the highest β-sheet and antiparallel contents among the tested formulations. This pattern may indicate that the intermediate PPHP level produced a greater apparent perturbation of the protein conformational distribution than the other formulations; however, this interpretation should be regarded as a hypothesis based on the FTIR-derived secondary-structure estimates rather than as direct evidence of the greatest conformational perturbation.

At higher PPHP concentrations, the progressively lower SPI concentration may reduce the extent of protein–protein interactions and alter the relative availability of protein and polysaccharide binding sites, thereby shifting the system toward a different structural equilibrium. Exposure of protein regions following partial unfolding may facilitate intermolecular association with PPHP polysaccharides through hydrogen bonding, hydrophobic interactions, and physical entanglement [37]. This interpretation agrees with the FTIR spectral broadening and shifts and with the SDS–PAGE evidence indicating formation of large SPI–PPHP complexes predominantly stabilized by non-covalent interactions. At higher PPHP levels (PPHP6–PPHP8), the partial recovery of α-helix content together with slightly lower β-sheet and antiparallel contents compared with PPHP4 suggested that conformational changes were not strictly concentration-dependent. With further PPHP incorporation, the accompanying reduction in SPI concentration may limit protein–protein association and reduce the extent of further protein unfolding or β-sheet formation. Meanwhile, the increasing proportion of PPHP may promote additional hydration and polysaccharide–polysaccharide association, potentially stabilizing an alternative molecular arrangement and accounting for the partial recovery of α-helices in PPHP6 and PPHP8. Rather, increasing PPHP may shift the system toward a new protein–polysaccharide equilibrium in which extensive polysaccharide association and reduced SPI concentration limit further protein–protein rearrangement. The changes in the FTIR-derived secondary structure may reflect the combined effects of PPHP incorporation and the accompanying reduction in SPI concentration. PPHP-derived polysaccharides may participate in hydrogen bonding and alter the hydration environment of SPI, while the lower protein concentration may independently influence the relative contribution of different secondary structural components. Collectively, the FTIR and secondary-structure results demonstrate that PPHP acts not merely as a passive filler but as a structural modulator of the SPI matrix, promoting protein conformational rearrangement and non-covalent protein–polysaccharide association. These molecular modifications provide a mechanistic link between PPHP incorporation and the enhanced rheological behavior, printing stability, and tunable texture of SPI-based 3D-printed gels.

### 3.7. In Vitro Gastrointestinal Digestibility

**Figure 7:**
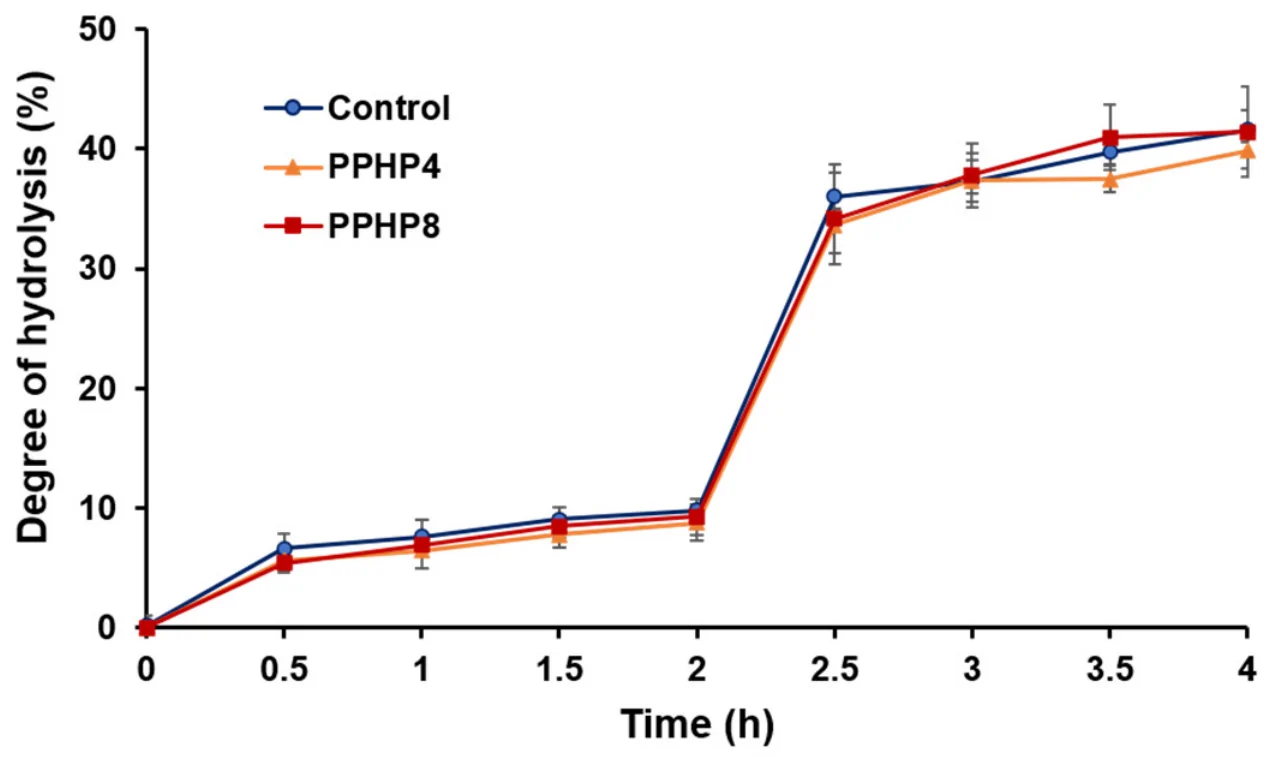
Degree of hydrolysis of SPI gels containing different concentrations of PPHP during in vitro gastrointestinal digestion, determined by the TNBS assay. The gastric digestion phase was conducted from 0 to 2 h, followed by the intestinal digestion phase from 2 to 4 h (*n* = 3).

The *in vitro* gastrointestinal digestibility of SPI–PPHP 3D-printed gels is shown in Figure 7. The DH increased progressively throughout the gastric and intestinal stages for all formulations, reflecting continuous breakdown of soy proteins into smaller peptides and amino acids [38]. At 0 min, DH was near zero for all formulations. DH rose gradually during the gastric phase, ranging from 8.80 ± 1.46% to 9.79 ± 1.00%, and then increased markedly after the transition to the intestinal phase. By the end of digestion (240 min), DH values were 41.59 ± 1.68%, 39.85 ± 1.52%, and 41.41 ± 3.78% for the Control, PPHP4, and PPHP8, respectively. Two-way ANOVA showed that digestion time had a significant effect on DH (*F*(8, 81) = 1040.20, *p* < 0.001), confirming the progressive increase in protein hydrolysis throughout digestion (Table S1). A significant main effect of formulation was also observed (*F*(2, 81) = 4.02, *p* = 0.022). However, the formulation × digestion time interaction was not significant (*F*(16, 81) = 0.54, *p* = 0.917), indicating that the changes in DH over digestion time did not differ significantly among the Control, PPHP4, and PPHP8 formulations. Although numerical differences were observed at some intermediate digestion times, particularly around the transition from the gastric to intestinal phase, these differences did not result in significantly different digestion trajectories among formulations. The significant main effect of formulation indicates that differences existed among the formulations when DH was considered across the digestion period as a whole; however, the absence of a significant formulation × time interaction indicates that PPHP incorporation did not substantially modify the temporal pattern of protein hydrolysis. The relatively similar final DH values among formulations further suggest that the magnitude of the formulation-related differences was limited under the tested digestion conditions. Therefore, the effects of PPHP on protein hydrolysis should be interpreted in terms of an overall formulation effect rather than a differential effect on the progression of digestion over time. This finding is consistent with previous reports showing that protein–polysaccharide complexes formed predominantly through non-covalent interactions, including hydrogen bonding, hydrophobic association, and electrostatic forces, generally do not substantially restrict enzymatic access to protein substrates, as covalent interactions between polysaccharides and soy protein isolate are largely absent [12,39]. This aligns with the SDS–PAGE results (Section 3.4), which similarly indicated that PPHP–SPI interactions were predominantly non-covalent rather than involving extensive cross-linking. In addition, Hu et al. [40] reported that incorporating polysaccharides, such as potato starch and guar gum, reduced the in vitro protein digestibility of soybean curd. This reduction was associated with weakened hydrophobic interactions and disulfide bonds among soybean proteins, accompanied by increased hydrogen bonding, which limited protein hydrolysis and the formation of small peptides and free amino acids during digestion. In contrast, lower concentrations of other polysaccharides did not significantly affect protein digestibility. More broadly, polysaccharides can influence protein digestibility in hybrid protein–polysaccharide hydrogels by acting as a physical barrier that reduces enzyme penetration, an effect linked to reduced enzyme diffusion and limited protein–enzyme contact area within the gel microstructure [41,42]. The lack of a significant DH reduction in the SPI–PPHP system may relate to the relatively open microstructure of PPHP-containing gels (Section 3.2), where interconnected pores and less densely packed regions likely facilitated enzyme penetration and diffusion, maintaining enzyme–substrate contact despite PPHP’s structural reinforcement. This microstructure–accessibility relationship offers a plausible explanation for the comparable DH profiles across formulations, though it should be interpreted cautiously, as enzyme diffusion and pore characteristics were not quantitatively measured in this study. Taken together, these findings indicate that the improved rheological and structural properties conferred by PPHP did not compromise protein digestibility. The reinforced protein–polysaccharide matrix remained sufficiently open to preserve enzymatic accessibility, demonstrating that PPHP can enhance the processing and printing performance of SPI-based 3D-printed foods without reducing their protein digestibility.

### 3.8. Viability of B. longum subsp. longum in PPHP-Containing Formulations

As shown in Table 4, the C-GAM exhibited the highest viable count (7.32 ± 0.09 log_10_ CFU/mL), which was significantly higher than those of all PPHP formulations (*p* < 0.05). Among formulations, PPHP6 exhibited the highest viability (7.16 ± 0.03 log_10_ CFU/mL), followed by PPHP8 (7.07 ± 0.02 log_10_ CFU/mL), with no significant difference between them; both were significantly higher than PPHP2 and PPHP4, while the Control (6.96 ± 0.03 log_10_ CFU/mL) was intermediate. PPHP2 showed the lowest viability (5.31 ± 0.02 log_10_ CFU/mL), significantly lower than all other treatments (*p* < 0.05). These results indicate a formulation-dependent rather than dose-dependent response of *B. longum* subsp. *longum* TBRC 7151 to partial replacement of SPI with PPHP. Relative to the Control, PPHP6 increased viability by 0.20 log units (approximately 1.6-fold). Although PPHP6 showed the highest viability among the PPHP-containing formulations (7.16 ± 0.03 log_10_ CFU/mL), its viable count remained significantly lower than that of the C-GAM control (7.32 ± 0.09 log_10_ CFU/mL; *p* < 0.05). These results indicate that PPHP6-containing formulations supported relatively high viability of *B. longum* subsp. *longum* compared with the other PPHP-containing formulations but did not support viability to the same level as the complete GAM medium under the conditions tested. This improvement may reflect the carbohydrate-rich composition of PPHP, alongside an improved balance between carbon and nitrogen sources as PPHP partially replaced SPI [43,44,45]. PPHP6 and PPHP8 performed similarly; however, without a statistically significant difference, PPHP6 cannot be considered superior to PPHP8. These mechanistic explanations remain hypothetical, as carbohydrate utilization, fermentation metabolites, phenolic composition, pH, water activity, and matrix structure were not directly measured—factors particularly relevant given the anaerobic nature of *B. longum* [46,47]. Moreover, probiotic viability also depends on the biopolymer type and processing technology used, which is relevant to the mechanistic arguments regarding hydrogen bonding, structural reinforcement, and protein–polysaccharide complexation [48]. Importantly, the present experiment was designed to evaluate the viability of *B. longum* subsp. *longum* in the tested formulations and did not include a recognized positive prebiotic control, such as fructooligosaccharides (FOS) or inulin. Consequently, the relative prebiotic activity of PPHP cannot be determined from the present data. In addition, viable counts alone are insufficient to establish prebiotic activity, which requires evidence of selective utilization by beneficial microorganisms and, ultimately, an associated health benefit. Therefore, the present findings should be interpreted as differences in *B. longum* viability among the tested formulations rather than evidence of a prebiotic effect of PPHP. Future studies should include an established prebiotic control, such as FOS or inulin, together with measurements of selective substrate utilization and fermentation metabolites to more appropriately evaluate the prebiotic activity of PPHP.

## 4. Conclusions

PPHP incorporation modified the rheological and printing properties of SPI-based inks for extrusion-type 3D food printing. Increasing PPHP levels generally increased apparent and complex viscosity, storage modulus (*G*′), deformation resistance, and structural recovery while maintaining shear-thinning behavior, improving post-deposition shape retention and dimensional accuracy. SEM and SDS–PAGE results indicated changes in protein–polysaccharide association and matrix organization, while FTIR suggested shifts in protein secondary-structure distribution. However, these analyses were qualitative or interpretative rather than definitive, as they did not confirm specific molecular interactions or covalent crosslinking or independently validate secondary-structure assignments. PPHP incorporation was associated with numerical reductions in hardness, gumminess, chewiness, and resilience, while springiness remained relatively stable, indicating that texture could be modulated without compromising structural stability. During simulated gastrointestinal digestion, DH increased substantially over time, with no significant formulation × time interaction, indicating that PPHP did not alter the temporal pattern of hydrolysis, although a significant overall formulation effect was detected. Viability of *B. longum* subsp. *longum* TBRC 7151 was formulation-dependent, with PPHP6 showing the highest viability among PPHP formulations but remaining below the C-GAM control, providing preliminary but not conclusive evidence of prebiotic support. Overall, PPHP shows potential as a fiber-rich co-ingredient for tailoring the rheological, structural, and textural properties of SPI-based 3D-printed foods, with PPHP8 offering the most favorable dimensional printing performance under the conditions tested.

## Figures and Tables

**Table 1:** Dimensional printing deviation and shape fidelity of printed sample.

Samples	Dimensional Printing Deviation (%)		Overall Dimensional Deviation (%)	Shape Fidelity (%) *
	Width	Height		
Control	4.25 ± 0.25 a	7.00 ± 0.75 a	5.63 ± 0.50 a	104.25 ± 0.25 a
PPHP2	3.50 ± 0.43 a	4.67 ± 0.52 b	4.08 ± 0.38 b	103.50 ± 0.43 a
PPHP4	1.92 ± 0.52 b	2.50 ± 0.43 c	2.21 ± 0.38 c	101.92 ± 0.52 b
PPHP6	1.17 ± 0.38 bc	0.83 ± 0.38 d	1.00 ± 0.33 d	101.17 ± 0.38 bc
PPHP8	0.25 ± 0.25 c	0.25 ± 0.25 d	0.25 ± 0.13 d	100.25 ± 0.25 c

Values are presented as mean ± SD (*n* = 5). Different letters indicate statistical significance (*p* < 0.05). * 100% = perfect match, >100% = expansion/spreading and <100% = shrinkage.

**Table 2:** Effect of PPHP concentration on the textural properties of 3D-printed SPI-PPHP gels.

Samples	Hardness (g)	Adhesiveness (g·s)	Springiness (mm)	Cohesiveness	Gumminess (g)	Chewiness (g·mm)
Control	2473.01 ± 460.24 a	−1.197 ± 0.263 a	0.667 ± 0.024 ns	0.488 ± 0.048 a	1196.68 ± 192.74 a	796.19 ± 120.47 a
PPHP2	1972.16 ± 441.83 a	−0.867 ± 0.400 ab	0.646 ± 0.081	0.449 ± 0.091 ab	946.99 ± 210.61 b	617.89 ± 109.83 b
PPHP4	1054.06 ± 376.21 ab	−1.075 ± 0.673 ab	0.676 ± 0.050	0.384 ± 0.034 b	381.71 ± 142.86 c	292.96 ± 98.11 c
PPHP6	1113.59 ± 130.02 ab	−2.050 ± 0.717 c	0.641 ± 0.029	0.344 ± 0.034 b	380.50 ± 24.56 c	244.01 ± 19.56 c
PPHP8	1373.10 ± 81.06 ab	−2.286 ± 0.475 c	0.641 ± 0.015	0.329 ± 0.022 b	451.54 ± 42.39 c	289.40 ± 28.31 c

Values are presented as mean ± SD (*n* = 10). Different letters indicate statistical significance (*p* < 0.05). ns = Not significant.

**Table 3:** Relative content (%) of protein secondary structures obtained from amide I spectral profile (after curve fitting).

Samples	α-Helix (1640–1670 cm^−1^)	β-Sheet (1620–1640 cm^−1^)	β-Turn (1670, 1620 cm^−1^)	Antiparallel (1680–1695 cm^−1^)
Control	36.94 ± 0.54 a	26.45 ± 0.031 c	19.29 ± 0.43	17.32 ± 0.62 b
PPHP2	32.21 ± 0.35 b	30.33 ± 0.20 b	19.13 ± 0.59	18.33 ± 0.71 b
PPHP4	28.10 ± 0.67 d	32.04 ± 0.41 a	18.41 ± 0.36	21.45 ± 0.95 a
PPHP6	29.91 ± 0.61 cd	30.55 ± 0.18 b	18.55 ± 1.02	20.99 ± 0.53 ab
PPHP8	30.00 ± 0.46 c	30.83 ± 1.23 ab	19.72 ± 0.70	19.45 ± 1.20 ab

Values are presented as mean ± SD (*n* = 3). Different lowercases showed significant differences between samples with and without cross-linking (*p* < 0.05).

**Table 4:** Viable counts of *Bifidobacterium longum* subsp. *longum* TBRC 7151 in formulations containing different concentrations of peach palm after incubation for 48 h under anaerobic conditions.

Treatment	Viable Count (log_10_ CFU/mL)
C (GAM)	7.32 ± 0.09 a
Control	6.96 ± 0.03 c
PPHP2	5.31 ± 0.02 e
PPHP4	6.65 ± 0.03 d
PPHP6	7.16 ± 0.03 b
PPHP8	7.07 ± 0.02 bc

Data are presented as mean ± SD (*n* = 3). C (GAM) represents the Gifu anaerobic medium broth control. Data are presented as mean ± SD (*n* = 3). Different letters indicate significant differences among treatments (*p* < 0.05).

## Data Availability

The original contributions presented in this study are included in the article/Supplementary Material. Further inquiries can be directed to the corresponding author.

## Supplementary Materials

The following supporting information can be downloaded at: https://www.mdpi.com/article/10.3390/foods15183336/s1, Figure S1: Curve fitting of amide I and secondary structure protein band assignment of SPI-PPHP gels at different PPHP concentration. Table S1. Two-way ANOVA results for the degree of hydrolysis (DH) of SPI–PPHP 3D-printed gels during in vitro gastrointestinal digestion.
