# Supplementary material for: Multifunctional Effects of Peach Palm (Bactris gasipaes) Heart Powder on the Printability, Structural Properties, Probiotic Viability, and Gastrointestinal Digestion of Soy Protein-Based 3D-Printed Foods
Source: Foods. 2026 Sep 20;15(18):3336. doi: 10.3390/foods15183336 (PMC13606209; doi:10.3390/foods15183336)
Supplement: Supplementary file 1 [file foods-15-03336-s001.zip › foods-4550938-supplementary.pdf]

Supplementary Figures and Tables

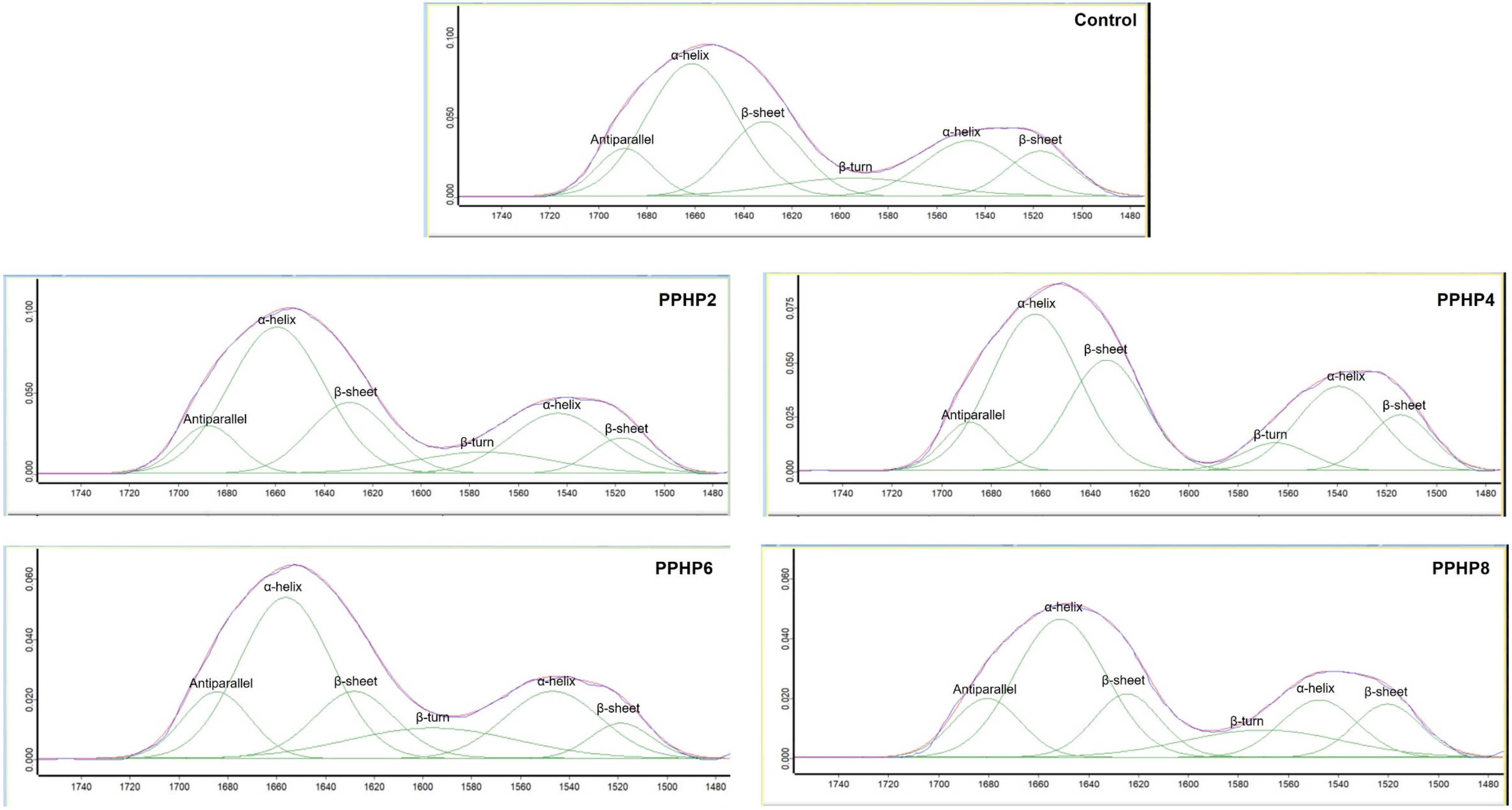

Figure S1. Curve fitting of amide I and secondary structure protein band assignment of SPI-PPHP gels at different PPHP concentration

Table S1. Two-way ANOVA results for the degree of hydrolysis (DH) of SPI–PPHP 3D-printed gels during *in vitro* gastrointestinal digestion.

Analysis of Variance

Source	DF	Adj SS	Adj MS	F-Value	P-Value
Treatment	2	27.2	13.59	4.02	0.022
Time	8	28147.4	3518.43	1040.20	0.000
Treatment*Time	16	29.2	1.83	0.54	0.917
Error	81	274.0	3.38		
Total	107	28477.8			

Coefficients

Term	Coef	SE Coef	T-Value	P-Value	VIF
Constant	20.347	0.177	114.97	0.000	
Treatment					
Control	0.525	0.250	2.10	0.039	1.33
PPHP4	-0.676	0.250	-2.70	0.008	1.33
Time					
0.0	-20.282	0.501	-40.52	0.000	1.78
0.5	-14.433	0.501	-28.83	0.000	1.78
1.0	-13.353	0.501	-26.68	0.000	1.78
1.5	-11.909	0.501	-23.79	0.000	1.78
2.0	-11.053	0.501	-22.08	0.000	1.78
2.5	14.264	0.501	28.50	0.000	1.78
3.0	17.125	0.501	34.21	0.000	1.78
3.5	19.036	0.501	38.03	0.000	1.78
Treatment*Time					
Control 0.0	-0.423	0.708	-0.60	0.552	2.37
Control 0.5	0.202	0.708	0.29	0.776	2.37
Control 1.0	0.069	0.708	0.10	0.923	2.37
Control 1.5	0.108	0.708	0.15	0.879	2.37
Control 2.0	-0.032	0.708	-0.05	0.964	2.37
Control 2.5	0.881	0.708	1.24	0.217	2.37
Control 3.0	-0.708	0.708	-1.00	0.320	2.37
Control 3.5	-0.209	0.708	-0.29	0.769	2.37
PPHP4 0.0	0.639	0.708	0.90	0.370	2.37
PPHP4 0.5	0.429	0.708	0.61	0.546	2.37
PPHP4 1.0	0.148	0.708	0.21	0.835	2.37
PPHP4 1.5	0.018	0.708	0.03	0.980	2.37
PPHP4 2.0	0.179	0.708	0.25	0.802	2.37
PPHP4 2.5	-0.306	0.708	-0.43	0.667	2.37
PPHP4 3.0	0.538	0.708	0.76	0.450	2.37
PPHP4 3.5	-1.223	0.708	-1.73	0.088	2.37
